# Commentary: Blockade of activin type II receptors with a dual anti-ActRIIA/IIB antibody is critical to promote maximal skeletal muscle hypertrophy

**DOI:** 10.3389/fphar.2018.00381

**Published:** 2018-04-19

**Authors:** Javier Durán, Manuel Estrada

**Affiliations:** Program of Physiology and Biophysics, Faculty of Medicine, University of Chile, Santiago, Chile

**Keywords:** bimagrumab, ActRIIA/B receptors, skeletal muscle, anabolic steroids, myostatin/activin

Balance in protein turnover influences the regulation of skeletal muscle mass (Schiaffino et al., 2013); it increases through anabolic processes and decreases owing to muscle wasting (as in diabetes and cancer), or muscle atrophy (as in aging and immobilization) (Basualto-Alarcón et al., 2014).

Endocrine and paracrine factors influence skeletal muscle growth by regulating different signaling pathways at the cellular and transcriptional levels. Transforming growth factor-β members, Myostatin and Activin A, which mediate signal transduction through the Smad2/3 have been suggested as negative regulators of muscle growth (Massagué, 2012).

Muscle mass increases owing to myostatin and activin inhibition via a soluble anti-ActRIIB ligand trap (Lee et al., 2005). Interestingly, in myostatin-null mice, treatment with anti-ActRIIB antibody causes additive muscle hypertrophy, suggesting that ActRII receptor ligands regulate muscle growth (Lee et al., 2005; Lach-Trifilieff et al., 2014).

Because both ActRIIA and ActRIIB receptors regulate muscle mass, researchers at Novartis Institutes for Biomedical Research investigated the effect of a blockade of both ActRIIA and ActRIIB receptors with human anti-ActRII antibody “Bimagrumab” (BYM338) on skeletal muscle growth in mice. They suggested that only synchronous inhibition of both receptors induces a maximal anabolic response (Lach-Trifilieff et al., 2014).

Morvan et al. reported the crystal structures of bimagrumab Fv complex with human ActRIIA and ActRIIB ligand-binding domains, indicating its similar binding with both receptors, although their epitopes share a 65% sequence identity. Nonetheless, bimagrumab has a 50-fold higher affinity for ActRIIB than for ActRIIA, probably owing to differences between several amino acid at the binding interface relevant for the interactions with bimagrumab (Morvan et al., 2017).

Since the blockade of ActRIIA or ActRIIB only partially blocks signaling downstream of myostatin and activin A stimulation, the effect of bimagrumab and its murine version (CDD866) was investigated on HEK293T/17 cells stably expressing Smad2/3; this effect was then compared with that of anti-ActRIIA or anti-ActRIIB antibodies. Inhibition of both receptors with a combination of these antibodies, bimagrumab, and CDD866, completely blocked Smad2/3 signaling induced by myostatin and activin A in comparison with the 30–50% inhibition with the either of the two antibodies.

To compare the effect of single or synchronous blockade of ActRIIA and ActRIIB on muscle growth, mice were treated with anti-ActRIIA, anti-ActRIIB, both antibodies, or bimagrumab. Treatment with a combination of anti-ActRIIA and anti-ActRIIB antibodies or bimagrumab increased muscle strength and mass by 20–30%, compared to the control, meanwhile, treatment with individual antibodies, increased by 10%. This suggested that higher growth response results from blockade of both receptors, compared to effects of individual antibodies.

Recently, both murine models of cancer cachexia and bone healing after osteotomy suggest that either CDD866 or bimagrumab protects against both body weight and muscle mass loss resulting from anti-cancer therapy, with no effects on bone fracture healing (Hatakeyama et al., 2016; Tankó et al., 2016). Furthermore, clinical studies report that bimagrumab treatment increases muscle mass and strength in sporadic inclusion body myositis (Amato et al., 2014) and sarcopenia (Rooks et al., 2017a), both recovery of thigh muscle volume and decline in intermuscular adipose tissue after casting (Rooks et al., 2017b), and an improvement in both lean and fat mass, and insulin sensitivity in patients with insulin resistance (Garito et al., 2018).

Morvan et al. reported the role of ActRIIA and ActRIIB in muscle growth, representing valuable targets for blockade in pharmacological studies, along with a potential therapeutic alternative for patients with decreased muscle strength and mass. The clinical trials resource on the NIH website (https://clinicaltrials.gov) indicates that the effect of bimagrumab was assessed in chronic obstructive pulmonary disease patients with cachexia and on weight loss in lung or pancreatic cancer patients; Bimagrumab increased muscle volume and is currently under clinical trials on patients who underwent hip fracture surgery or in patients with sarcopenia, overweight, and obesity, with type 2 diabetes.

Since muscle loss is also associated with significant reductions in anabolic hormones, we investigated the effects of androgens on cardiac and skeletal muscle cells. Myostatin decrease muscle mass, and influence bone health after androgen deprivation in mice; this was assessed through administration of soluble ActRIIB ligand traps (Koncarevic et al., 2010; Pan et al., 2016). Furthermore, myostatin levels are negatively regulated by androgens (Mendler et al., 2007) and in men, testosterone declines with age, suggesting that low plasma levels can cause or accelerate muscle- and age-related diseases. Some myostatin/activin members are upregulated with age (Brun and Rudnicki, 2015); however, a recent study suggests the opposite, thereby presenting new changes regarding atrophic agents and loss of skeletal muscle mass (Schafer et al., 2016).

Physiological circulating levels of testosterone are important for ongoing muscle functions, but high concentrations induce cardiac and skeletal muscle hypertrophy (Basualto-Alarcón et al., 2014). Currently, meta-analysis and clinical trials show that low levels of testosterone are associated with metabolic syndrome, obesity and cardiovascular diseases (Wang et al., 2011). Because testosterone is a key physiological anabolic hormone, decreased plasma concentrations should be considered a primary cause for muscle loss and sarcopenia.

Under normal states, catabolic processes are balanced with anabolic processes; hence, it would be interesting to investigate the relationship between activation of anabolic pathways and inhibition of catabolic pathways to prevent muscle atrophy or to maintain and increase muscle mass. Myostatin levels negatively correlate with diverse muscle growth modulators (as insulin-like growth-factor-1 and growth hormone) that consequently potentiate myostatin/activin ligand atrophy (Schwarz et al., 2016). The pathways underlying hormonal regulation of muscle protein metabolism are complex and multifactorial. Anabolic hormones regulate protein synthesis via transcriptional and translational regulation through the Akt-mTORC1 pathway, which is inhibited by Smad2/3 (Sartori et al., 2014).

Hence, important aspects regarding interactions among different muscle growth regulators and drugs that inhibit ActRIIA/IIB receptors warrant further investigation. Pharmacological and preclinical studies aimed to identify integrated regulatory mechanisms that operate on muscle metabolism and growth could be used to promote healthy muscle adaptation and delay muscle loss associated with progression of muscle-related diseases and aging.

## Author contributions

All authors listed have made a substantial, direct and intellectual contribution to the work, and approved it for publication.

### Conflict of interest statement

The authors declare that the research was conducted in the absence of any commercial or financial relationships that could be construed as a potential conflict of interest. The reviewer JC and handling Editor declared their shared affiliation.
